# Robust Computationally-Efficient Wireless Emitter Classification Using Autoencoders and Convolutional Neural Networks

**DOI:** 10.3390/s21072414

**Published:** 2021-04-01

**Authors:** Ebtesam Almazrouei, Gabriele Gianini, Nawaf Almoosa, Ernesto Damiani

**Affiliations:** 1 Emirates ICT Innovation Centre, Khalifa University of Science and Technology, Abu Dhabi 127788, United Arab Emirates; gabriele.gianini@unimi.it (G.G.); nawaf.almoosa@ku.ac.ae (N.A.); ernesto.damiani@ku.ac.ae (E.D.); 2 Department of Electrical Engineering and Computer Science, Khalifa University of Science and Technology, Abu Dhabi 127788, United Arab Emirates; 3 Dipartimento di Informatica, Università degli Studi di Milano, 20133 Milano, Italy; 4 Research Centre on Cyber-Physical Systems (C2PS), Khalifa University, Abu Dhabi 127788, United Arab Emirates

**Keywords:** Deep Learning, Denoising Autoencoders, convolutional neural networks, classification, IEEE Wi-Fi protocols, LTE, spectrum management

## Abstract

This paper proposes a novel Deep Learning (DL)-based approach for classifying the radio-access technology (RAT) of wireless emitters. The approach improves computational efficiency and accuracy under harsh channel conditions with respect to existing approaches. Intelligent spectrum monitoring is a crucial enabler for emerging wireless access environments that supports sharing of (and dynamic access to) spectral resources between multiple RATs and user classes. Emitter classification enables monitoring the varying patterns of spectral occupancy across RATs, which is instrumental in optimizing spectral utilization and interference management and supporting efficient enforcement of access regulations. Existing emitter classification approaches successfully leverage convolutional neural networks (CNNs) to recognize RAT visual features in spectrograms and other time-frequency representations; however, the corresponding classification accuracy degrades severely under harsh propagation conditions, and the computational cost of CNNs may limit their adoption in resource-constrained network edge scenarios. In this work, we propose a novel emitter classification solution consisting of a Denoising Autoencoder (DAE), which feeds a CNN classifier with lower dimensionality, denoised representations of channel-corrupted spectrograms. We demonstrate—using a standard-compliant simulation of various RATs including LTE and four latest Wi-Fi standards—that in harsh channel conditions including non-line-of-sight, large scale fading, and mobility-induced Doppler shifts, our proposed solution outperforms a wide range of standalone CNNs and other machine learning models while requiring significantly less computational resources. The maximum achieved accuracy of the emitter classifier is 100%, and the average accuracy is 91% across all the propagation conditions.

## 1. Introduction

The rapidly growing mobile traffic and user base is fueling the demand for spectrum resources that are increasingly challenging to provision due to scarcity and access restrictions. One of the mitigation strategies consists of improving spectrum utilization efficiency by sharing the unlicensed operation of radio-access technologies (RATs). Recent examples include the use of the 5 GHz band, which Wi-Fi occupies (IEEE 802.11a) and an unlicensed version of Long-Term Evolution cellular standard (unlicensed-LTE) [1,2], as well as the unlocking of the 6 GHz band for Wi-Fi 6 (IEEE 802.11ax) and 5G New Radio Unlicensed (NR-U) standards that will operate along with incumbent primary users [3].

Realizing the benefits of the shared unlicensed operation to meet the increasingly stringent quality of service (QoS) application targets is contingent upon meeting challenging requirements, which include ensuring fair and harmonious coexistence between users, secure access in line with regulations, and maximal performance through optimized resource allocation and interference management. The challenge in meeting these requirements emanates from the complexity of the emerging access environments, which are influenced by a confluence of physical (channel effects), human (user access and mobility patterns), and technological (design and *modus operandi* of RATs) factors.

A potential solution is advancing RAT intelligence to equip radio assets with adaptive learning and decision-making capabilities that will enable a greater level of autonomous operation compared to centralized schemes [4]. Intelligent spectrum monitoring is arguably a core component of adaptive radio learning that enables RATs to collect measurements and make sophisticated real-time inferences about the spectral state that drive reasoning and intelligent decision-making. This has motivated several works that build on two enabling technologies: The first is the software-defined radio (SDR), allowing programmable radio frequency (RF) operation across a wide range of frequency bands and with diverse cost and form-factor options [5]. The second consists of Artificial Intelligence and Machine Learning (AIML) techniques, whose performance and sophistication have increased, especially in the area of Deep Learning (DL) [6]. DL allows learning hierarchical representations of discriminant features in a generalized and efficient manner compared with the intensive and rigid feature engineering by human experts [7]. The introduction of Machine Learning techniques in communication is detailed in [8]. Many researchers have investigated using DL for wireless communication [9,10,11,12,13], some of them proposing DL models for different applications than signal processing [14,15,16,17,18]. Detailed surveys about DL for mobile and wireless networking are provided in [19,20].

Applying DL to wireless communication is currently an active research area (as highlighted in [19,21,22] and the references therein); this is motivated by similarities with other domains of successful DL application, such as speech and object recognition [23], and by the ability to build size-appropriate training data sets as wireless networks inherently generate large data volumes that can be efficiently collected and ingested [24].

### 1.1. Related Work

Existing intelligent spectrum works range from radio observatories and test-beds that monitor and provide high-level insights on a large geographical scale [25,26,27,28], to systems for radio anomaly detection and device fingerprinting [29,30,31,32,33], and various aspects of waveform classification [9,10,34,35,36,37,38,39].

Several works are concerned with AIML-based emitter RAT ( also referred to as *protocol*, *wireless standard*, and *wireless technology* classification) classification, which act as RAT-agnostic data-driven detectors that can be used for accurate access pattern detection and prediction of RATs operating in unlicensed shared bands. As the medium access schemes for unlicensed, shared RATs are mainly envisioned as variants of Listen-Before-Talk (LBT) schemes [40], where spectrum access is controlled by schemes based on sensing spectrum occupancy, RAT classification is the primary driver potentially for optimizing spectrum utilization and minimizing interference through intelligent and situation-aware dynamic spectrum access. It also can be utilized as a tool to support access policy enforcement for automated detection of violations that is more coverage efficient than manual human-resource based in-field analysis [41,42].

Emitter classification works include proposals based on feature engineering [43,44,45], as well as DL-based proposals using time-frequency (TF) representations that include spectrograms calculated using the Short-time Fourier Transform (STFT), as well as other custom TF representations [11,41,46,47,48,49,50]. Compared to strictly time-based features, TF representations can lead to better performance in emitter classification [51] and allow visualizing rich multi-emitter scenarios as patterns recognizable by human domain experts [46]. Formulating emitter classification as an object recognition problem allows leveraging state-of-the-art DL algorithms mainly based on training supervised convolutional neural network (CNN) classifiers which achieved top performance in other application domains [52].

However, there are limitations associated with CNN-based emitter classification. First, the visual patterns in TF representations are susceptible to corruption induced by the communication channel, which can severely degrade classification performance. Recent works showed that the visual features in spectrograms—the archetypal TF representations—could be indiscernible in low SNR conditions and significantly altered by frequency-selective fading [41,47]. The degradation can be significantly more pronounced in harsh channel conditions encountered in typical indoor environments due to severe multipath and non-line-of-sight (NLOS) conditions and mobility-induced Doppler effects. Second, the performance gains of CNNs might come at a high computational cost. While an abundance of computing resources might be available at the training phase of CNNs, the resulting inference engines may be deployed settings such as network edge [53] that are constrained in terms of computational resources and energy consumption and favor tight coupling between the RF circuits (sensing component) [54,55,56]. Unless addressed, the high computation and energy cost of CNNs might be a significant limiting factor towards broader adoption.

### 1.2. Contributions of Paper

The aforementioned challenges highlight the need for improving the robustness and the computational efficiency of TF-based emitter classification. This work proposes a novel emitter classification approach that uses a hybrid DNN consisting of a convolutional denoising autoencoder (CDAE), followed by a CNN classifier. By construction, in this approach, the representation learning phase devoted to denoising (performed by the CDAE) and the classification phase (performed by the CNN) are performed separately.

The theoretical motivation behind this approach relies on the following considerations: (1) the decoupling allows to perform a more efficient training and (2) a standalone representation learning phase focused on obtaining a reconstructed TF image before classification can more effectively support the CNN. The classifier can operate based on more clear-cut visual features of the TF representation after the representation learning. In a sense, the use of the CDAE allows incorporating explicitly into the process the priors about the protocol spectrogram original visual features that have been degraded during the signal propagation.

In practice, we demonstrate by simulation that, compared to the state-of-the-art standalone CNNs, our hybrid DNN approach achieves high accuracy under harsh propagation conditions. Our proposed DNN requires significantly less computational resources compared to standalone CNNs. The main contributions of the work are summarized as follows:

- A CDAE is trained to reconstruct the original visual patterns of pre-channel emissions out of spectrograms corrupted by harsh channel conditions. We conducted a comprehensive study in reconstruction performance using simulation results for several standards, including LTE and versions of Wi-Fi, under harsh propagation conditions that include SNR, multipath, NLOS, and Doppler.
- A CNN performs emitter classification using the denoised representation generated by the CDAE, leading to high classification accuracy under harsh channel conditions. Moreover, the resulting CNN requires significantly less computational resources as it operates on a compressed representation with lower dimensionality than spectrogram-fed CNN classifiers. We demonstrate, using simulations, that our proposed hybrid CDAE-CNN approach outperforms in classification accuracy and computational cost a wide range of DNN and ML-based schemes while requiring significantly less computing resources.

The rest of the paper is structured as follows. Section 1.1 presents the related work about DL for wireless communication. Section 2 describes our system model and the problem statement. In Section 2, simulation setup and data generation for unlicensed LTE and Wi-Fi standards are explained. Section 3 details our DL-based approach to classify wireless signals operating in the same unlicensed band. In Section 4, we report results under various noise scenarios, while the comparison between our approach and other ML and DL algorithms is presented in Section 5. Conclusions are drawn in Section 6.

## 2. System Model

**Figure 1:**
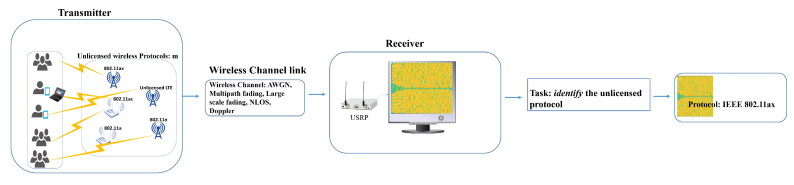
Emitter classification system model.

Consider the setting illustrated in Figure 1, which involves *N* wireless devices, each operating a Radio Access Technology (RAT) drawn from a set of *M* distinct RAT types, and a single receiver, tasked with monitoring device spectrum activity. Denote the devices by $d_{nm}$, where n=1,⋯,N is an index reflecting a unique usage context that includes user activity patterns and application data profiles, and by m=1,⋯,M the RAT-type label. When in transmission mode, $d_{nm}$ emits an *upconverted* version of a base-band time-varying signal $s_{nm}\left(t\right)$ by Radio Frequency (RF) with a given carrier frequency. The signal $s_{nm}\left(t\right)$ contains payload data formatted into RAT-defined packets, and its bandwidth and center frequency determine the spectrum band of operation, which can be fixed or dynamically determined as is the case with the unlicensed shared operation. A typical implementation of the receiver uses software-defined radios (SDRs), which are available off-the-shelf or custom-made [57] for applications including radio-environment maps (REMs) that characterize spatio-temporal spectrum access patterns and provide a useful tool for enforcing access policies by identifying violations. Assuming that there is no interference and that the receiver is tuned to the same spectrum band as the emitter, the *downconverted* received signal is denoted as $r(s_{nm})$ and can be modeled as

(1) $$r\left(s_{nm}\right)=H\left(s_{nm}\right)+G\left(s_{nm}\right),$$

where $G(s_{nm})$ denotes the additive white Gaussian noise (AWGN), and $H(s_{nm})$ other physical and user-induced degradation present in the wireless communication link between $d_{nm}$ and the receiver: it may include multipath, shadowing and non-line-of-sight (NLOS), mobility-induced Doppler effects, and specific Signal-to-Noise Ratio (SNR) conditions. Upon reception, $r(s_{nm})$ is converted to a spectrogram by applying the Short-Time Fourier Transform (STFT) [57]. We selected the spectrogram mainly because it is one of the most general and commonly-used time-frequency representations. The model and the methodology we employ can be extended to other representations, e.g., to spectrogram’s functions, such as the power spectrum transform and other custom time-frequency representations.

Let us denote by $R\left(s_{nm}\right)\in C^{N_{f}\times N_{t}}$ the matrix of complex values representing the the STFT of the received signal $r(s_{nm})$, with matrix elements $R\left(s_{nm}\right)\left(\tau ,\omega \right)$, where τ and ω denote values of the discretized time and frequency, respectively, while $N_{f}$ and $N_{t}$ denote the number of points sampled along the frequency dimension and time dimension, respectively. If we denote by $i\in [1,N_{f}]$ and $j\in [1,N_{t}]$, respectively, the index of the time and of frequency discretized values, the elements (pixels) of the $N_{f}\times N_{t}\;$ matrix are denoted by ${\left[R\left(s_{nm}\right)\right]}_{ij}$.

The real valued spectrogram $R\left(s_{nm}\right)\in R^{N_{f}\times N_{t}}$ of the received signal is obtained by calculating pixel-by-pixel the norm of the corresponding complex number in the spectrogram $R(s_{nm})$, i.e.,

$${\left[R\left(s_{nm}\right)\right]}_{ij}\equiv \left|{\left[R\left(s_{nm}\right)\right]}_{ij}\right|$$

Summarizing the relationships between the defined quantities we have

$$s_{nm}\left(t\right)\overset{H+G}{\to }r\left(s_{nm}\left(t\right)\right)\overset{STFT}{\to }R\left(s_{nm}\right)\in C^{N_{f}\times N_{t}}\overset{norm}{\to }R\left(s_{nm}\right)\in R^{N_{f}\times N_{t}}$$

The chain of transformations applied to $r(s_{nm}\left(t\right))$ can be applied to the original transmitted signal $s_{nm}\left(t\right)$. We use the following notation conventions:

$$s_{nm}\left(t\right)\overset{STFT}{\to }T\left(s_{nm}\right)\in C^{N_{f}\times N_{t}}\overset{norm}{\to }T\left(s_{nm}\right)\in R^{N_{f}\times N_{t}}\;$$

The comparison between the transmitted signal real valued spectrogram $T(s_{nm})$ and the received signal real valued spectrogram $R(s_{nm})$ is at the heart of the training of the Denoising Autoencoders described in the next section.

Notice that sometimes, to reduce the dimensionality of the problem, the values of each spectrogram are encoded to binary values using a comparison to the average value of the pixel. For instance, one can define

(2) $${\left[B\left(s_{nm}\right)\right]}_{ij}\equiv \left\{\begin{array}{cc} 1 & if\;{\left[R\left(s_{nm}\right)\right]}_{ij}>\langle R(s_{nm})\rangle \\ 0 & otherwise \end{array}\right.$$

so that $B\left(s_{nm}\right)\in {\left\{0,1\right\}}^{N_{f}\times N_{t}}$.

In this work, we assume that each spectrogram captures a single emission of each of the active RAT types. This assumption is motivated by the common practice of programming SDRs to scan multiple frequency bands [25,28]. Our model can be expanded upon to include wideband spectrograms that can span multiple emissions by using spectrum localization [46], and other time-frequency representations based on sweeping carrier frequency [58].

**Figure 2:**
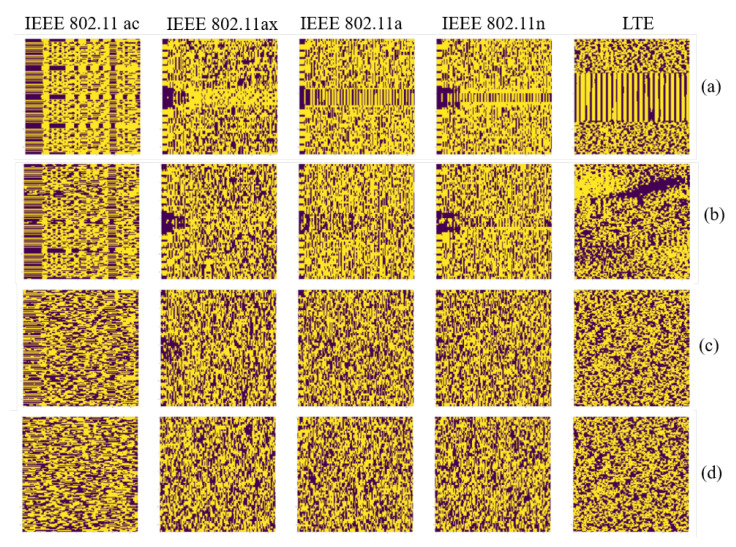
Spectrograms for different IEEE 802.11 protocols and unlicensed LTE signals: (**a**) the transmitted spectrograms, the received spectrograms for (**b**) *Scenario 2* and SNR 20 dB, (**c**) *Scenario 1* and SNR 5 dB, and (**d**) *Scenario 1* and SNR 0 dB.

Having calculated the spectrogram $R(s_{nm})$, and possibly its binarized version $B(s_{nm})$, the receiver passes it to the emitter classification algorithm that outputs an estimate of the operating RAT type. Figure 2 shows samples of the received spectrograms for some combination of channel scenarios and SNR levels. The transmitted spectrograms for each IEEE 802.11 protocol and LTE are presented in the first row of Figure 2. The LTE spectrogram could be corrupted easily under *Scenario 2* for SNR 20 dB. Channel *Scenario 1* and SNR 0 dB damage the preamble’s visual pattern in the captured spectrograms. The detailed description about data generations and the implemented Channel *Scenarios* are discussed later in Section 4. In real environments, it is hard to know the channel condition and to model it by a precise equation, especially in a harsh dynamic environment. To perform the identification task and have a system with a robust and high accuracy, the problem of the channel and noise effects in the received spectrograms should be solved. In the following section, our proposed methodology to achieve a robust identification task is detailed.

## 3. Proposed System and Methodology

The proposed approach for protocol identification consists of two main phases: (1) *Signal Denoising*: Reconstructing the unlicensed signals by removing the noise and the signal degradation effects using the DL model. (2) *Protocol Identification*: Identifying the unlicensed bands for the corrupted signals based on denoising DL weights.

**Figure 3:**
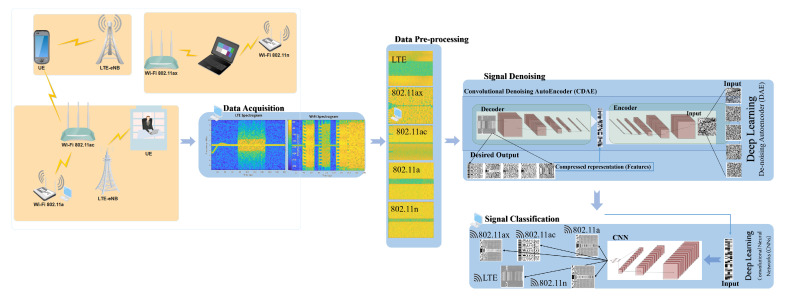
Proposed system for unlicensed protocol identification under noise and different channel propagation models.

To study the proposed system’s performance, first, a simulation environment is developed for LTE and WLAN 802.11 standards. Second, the data are generated and collected under different SNR values and channel propagation models. Third, data preprocessing is performed to prepare the DL model’s data by mapping the signals to the corresponding spectrograms. Fourth, the signal denoising is performed for each dataset to reconstruct the corrupted received signal spectrogram for each protocol using Convolutional Denoising Autoencoders (CDAEs). Finally, a signal classification is performed to identify the received protocol using CNN classifier model taking into input the CDAE representation. Figure 3 illustrates the proposed system for using DL.

### 3.1. Signal Denoising

The data denoising phase of our approach is based on particular artificial neural network layered architectures known as Denoising Autoencoders (DAE) [59], which are typically used to reconstruct data from a corrupted input.

In standard Autoencoders (AE), the input is received under the form of examples, and AE is trained to reconstruct them, while the DAEs input comes under the form of noisy examples, and the objective for DAE is to reconstruct their original non-noisy form (both non-noisy and noisy examples are provided to the DAE). In radio wireless communication, examples can be obtained either by measuring the target output $T(s_{nm})$ and the input $R(s_{nm})$ in a physical environment or from a radio simulation environment, or by artificially corrupting non-noisy data.

Autoencoders (AEs) are DL models used for self-supervised learning of an encoding of the input data. The input data instance can be an image or any other signal. The input is fed to the AE in the form of a 1D or a multidimensional array (e.g., a 2D image) typically flattened into a 1D form. Hereafter, we denote this array by $x=(x_{1},\cdots ,x_{k},\cdots ,x_{K})$ (where *K* is the total number of elements in the flattened array). AEs learn to encode an input data set in a compact representation that preserves the statistical correlation properties of the original distribution of inputs most relevant for the reconstruction. The AE architecture consists of one or more encoding layers and of as many decoding layers. A special role is assigned to the central (hidden) layer, called *code layer*. The main role of the encoding layers is to map the input vector *x* into the hidden representation $y=f_{\theta }\left(x\right)$; when there is a single encoding layer, *f* is defined by the following matrix equation:

(3) $$f_{\theta }\left(x\right)=\sigma \left(Wx+b\right)$$

where θ is a shorthand for (W,b) and represents the set of parameters defining *W* and *b*, *W* is the weight matrix, and *b* is the offset vector, while σ defined as a nonlinear function acting on the matrix elements and can be a Rectified Linear Unit (ReLU) function or a sigmoid function. With more encoding layers, the computation is iterated from one layer to the next.

The mapped representation *y* is decoded to get the reconstruction matrix *z*. The size of matrix *z* is the size of the input *x*. The reconstructed matrix $z=g_{\theta^{\prime }}\left(y\right)$ with a single decoding layer, is defined as

(4) $$g_{\theta^{\prime }}\left(y\right)=\sigma \left(W^{\prime }y+b^{\prime }\right)$$

With more decoding layers, the computation is iterated from one layer to the next. The value of the parameters *W*, $W^{\prime }$, *b*, and $b^{\prime }$ is passed to a loss function during the learning process. The parameter values can be optimized by minimizing the loss function. There are different loss functions which can be selected to train the AE parameters: usually for binary data, the loss function is the binary cross entropy (BCE), which is typically used for binary classification.

(5) $$BCE=-\sum_{k=1}^{K}\left[x_{k}log\left(z_{k}\right)+\left(1-x_{k}\right)log\left(1-z_{k}\right)\right]$$

where *K* is the total number of input array elements. Mean Square Error (MSE) is also a common loss function that is always non-negative:

(6) $$MSE={\frac{1}{K}\sum_{k=1}^{K}\left|\right|\left(x_{k}-z_{k}\right)\left|\right|}^{2}$$

We used the MSE as a loss function for the reconstruction error in our DAE, because it is known to work better with the DAE in image reconstruction. In our model, the MSE loss function is minimized by the *Adaptive Gradient Descent* (AGD).

DAEs use a noisy input $\hat{x}\neq x$ and are trained to produce encodings that restore the properties of the original non-noisy input. In our case, the noisy input is $\hat{x}=R\left(s_{nm}\right)$, the real valued spectrogram of the received signal, while the target output is $x=T(s_{nm})$) the real valued spectrogram of the transmitted signal. During the training of the DAE, a reconstructed spectrogram *z* obtained from the noisy signal spectrogram $\hat{x}$ is compared to the non-noisy target *x*. The non-noisy and noisy version of each input are obtained either by measuring the $T(s_{nm})$ and $R(s_{nm})$ signals in a physical environment or from a radio simulation environment, or even by artificially corrupting non-noisy data.

**Figure 4:**
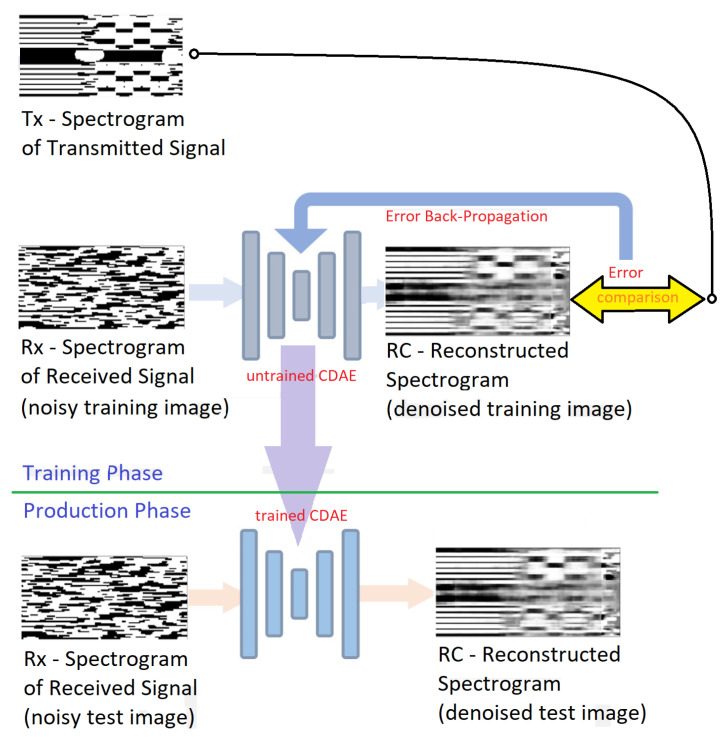
Schematic view of the operation for signal denoising using a Convolutional Denoising Autoencoder (CDAE). The input is noisy data (spectrograms of the received signal under various channel propagation scenarios). The encoder and decoder consist of multiple convolutional layers: the output is compared to the clean signal’s spectrogram. By optimizing the loss function, the network finds a denoised representation [62].

In our proposed DAE model, convolutional layers are used where the parameters *W* and *b* of each images batch are shared among all the locations to provide spatial locality. In this way, we put together the advantages of DAEs and the low complexity of the CNN paradigm. The resulting architecture is a Convolutional Denoising Autoencoder (CDAE). In general, CDAEs are very effective in signal processing [60] and in image processing perform better than classical DAEs [61]. Figure 4 shows the process of the proposed CDAE for signal denoising.

The effectiveness of using DAEs for reconstructing spectrograms of corrupted signals in unlicensed bands had been reported in our recent works [62,63]. However, it was only applied for denoising two Wi-Fi protocols in light noise scenarios. In this paper, we expanded our analysis substantially, using CDAEs to denoise multi-protocol signals, including unlicensed-LTE, in view of performing protocol identification. Furthermore, we assessed the effectiveness of CDAE for various propagation models and SNR values, with severe degradation in signal reception in harsh environments. We tuned the CDAE architecture so that it *generalizes* with consistent performance across all signals operating in the unlicensed spectrum and all noise levels and harsh environments. The architecture of our CDAE is explained in detail in Section 4.4.

### 3.2. Protocol Identification

We aim to apply ML in identifying the unlicensed radio technology specifically for spectrum sharing. Some signal processing functions can be learned within the physical layer as discussed in [13]: ML is used only for modulation classification of single-carrier modulation schemes using the CNN on radio frequency time-series data [13]. There are other ML methods applied for classifying radio signals such as SVMs [44], small feed-forward neural networks, and random forests [64].

We are now ready to discuss the protocol identification stage to be deployed after the CDAE stage for signal denoising. The architecture of our classifier is a Convolutional Neural Network, fed with with the features learnt by the CDAE in signal denoising process. The basic block of any CNN is the convolution, a simple application of a *filter* to an input that results in an activation. CNN filters are locally connected to capture correlations between different data regions in the image and output a feature map. The convolutional structure reduces the number of model parameters significantly and provides a robust recognition of affine invariance [6]. Powerful CNN models developed for imaging applications include ResNet, Inception-V4 [65], and GoogLeNet [66]. These models mostly differ from one another in terms of the CNN depth (Different inception and residual techniques were also proposed to overcome overfitting and gradient vanishing problems that are typical of “deep” CNNs. A detailed discussion of these techniques is outside the scope of this paper.).

CNNs were also adapted for video action recognition using 3D CNNs [67]. While our intuition supported the notion that CNNs could perform well on the snapshots/images of wireless spectra, we did not jump to conclusions. Instead, we implemented several ML models and compared their protocol identification performance across different SNR values and propagation models.

**Figure 5:**
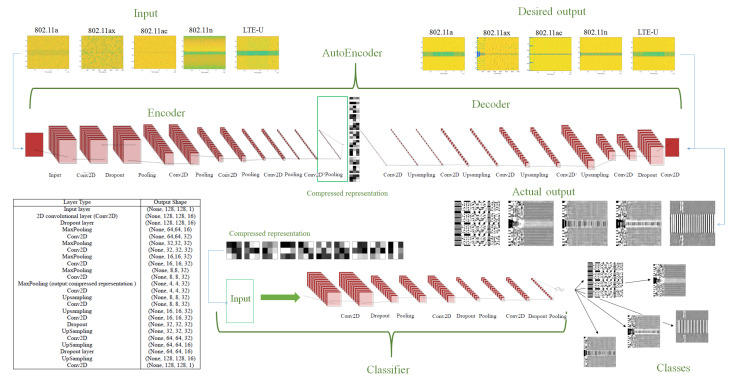
Signal classifier architecture composed of multiple convolutional layers. The classifier’s input is the compressed representation of the denoising autoencoder, and the output is the class.

Our resulting pipeline, which composes a DAE and a CNN classifier, includes a series of convolutional layers, a *maxpooling* layer, a fully connected layer, *dense* layer, and a *softmax* activation layer to perform the classification. The input to our CNN-based classifier is the DAE weights’ matrix (compressed representation), and the output is the class type of the signal. Our overall architecture for signal classification is illustrated in Figure 5. A further detailed explanation is given in Section 4.5.

## 4. Experiments and Results

### 4.1. Dataset Generation for LTE and IEEE 802.11 Family

We focused on Wi-Fi signals operating in the 5 GHz industrial, scientific, and medical (ISM) band in our experimentation. The selection of this unlicensed band is due to LTE, which can operate in the 5 GHz band, based on the operators’ preference [68]. To the best of the authors’ knowledge, there is no available dataset for wireless local area network (WLAN) 802.11 protocols or LTE, especially under multiple noise scenarios and propagation models; therefore, we resorted to data generated through simulation. We set up the simulation of the following five protocols operating in ISM bands: LTE, IEEE 802.11ac, IEEE 802.11ax, IEEE 802.11n, and IEEE 802.11a under different channel conditions. Protocols and conditions will be further detailed in Section 4.2. IEEE 802.11ax is the high throughput and efficiency WLAN amendment, which will replace both IEEE 802.11n and IEEE 802.11ac [69,70]. The end-to-end radio signal environment is built using the MATLAB WLAN System Toolbox for various WLAN 802.11 standards such as IEEE 802.11a, IEEE 802.11n, IEEE 802.11ac (Wi-Fi5), and IEEE 802.11ax (Wi-Fi6). MATLAB also includes LTE System Toolbox, which is used to build, design, and generate LTE waveforms and model end-to-end LTE communication links. The propagation of channel modeling functions allows fading and noise to the transmitted LTE and WLAN signals. In the following subsections, the implementation system for WLAN IEEE 802.11 protocols and unlicensed LTE is explained. The simulation setup details for LTE and IEEE 802.11 standards, the characteristics, the channel conditions, and the received signal for each protocol or RAT are all detailed in Appendix B.

### 4.2. Dataset Preparation

The simulation for each IEEE 802.11 protocol and LTE standard has been run independently to generate signals and save the radio spectrogram images for $T(s_{nm})$ and $R(s_{nm})$, under various channel scenarios and SNR values. The radio spectrogram images correspond to as many preambles for each IEEE 802.11 protocol and LTE subframes.

Each spectrogram represents the *Short-Time Fourier Transform*(STFT) [57] of the raw time series $s_{nm}\left(t\right)$ corresponding to the signal of a preamble. Spectrograms were selected as signal representations because of their ability to capture the behavior of multiple received signals. Each spectrogram image represents the STFT for transmitted or received signal as a function of the (discretized) time τ and frequency ω. Each spectrogram image consists of 64×3782 pixels where 3782 time intervals are captured, and for each interval, 64 frequencies are computed. To reduce the high dimensionality in the spectrograms, they were binarized according to Equation (2).

### 4.3. Datasets

We generated 25 datasets, representing as many combinations of channel scenarios (5 scenarios) and SNR values (5 levels: 20 dB, 15 dB, 10 dB, 5 dB, 0 dB). Each dataset is evaluated under a specific channel scenario and SNR level. In total, we generated 500,000 spectrograms images. Each of our 25 datasets consists of 20,000 spectrogram images, i.e., 10,000 pairs of transmitted and received spectrograms, 2000 for each of the five protocols we considered: IEEE 802.11a, IEEE 802.11ac, IEEE 802.11ax, IEEE 802.11n, and LTE. A summary of the implemented noise model scenarios for LTE signals is given in Table 1. The characteristics, the channel conditions, and the received signal for each protocol/RAT are detailed in Appendix A.

Figure 2 shows samples of the captured spectrograms for some combination of channel scenarios and SNR levels.

### 4.4. Results of Autoencoder-Based Denoising

The dataset for each channel scenario was examined independently to evaluate our Convolutional Denoising Autoencoder (CDAE) performance in reconstructing the clean spectrograms. Overall, 10,000 transmitted spectrograms make up the clean dataset, while 10,000 received spectrograms make up the noisy dataset. Each spectrogram image is resized to 128×128.The dataset was split, 80% for training and the remainder for testing the model.

#### 4.4.1. The Proposed Autoencoder Architecture

The structure of our CDAE consists of 24 layers: an input layer, eleven 2D layers for the *encoder* part, and twelve 2D layers for *decoder* part. The 2D *encoder* convolutional layers consists of a *rectified linear units (ReLU)* activation function layer, a *dropout* layer, and a 2D *max-pooling* layer. The *convolutional* layer *C* is used to learn the weights and biases of the spectrogram parameters. The *ReLU* layer computes the max(0,x) element-wise activation function thresholding at zero. The *dropout* layer is utilized for better generalization and to avoid overfitting. *Maxpooling* is performed for downsampling the convolutional layer *C* to lower the spatial dimensions.

The input data of the *encoder* consist of a quantized spectrogram image with a size of (128, 128). The first hidden layer C1 is a convolutional layer consists of 16 feature maps. Each feature map is connected to a kernel where the size of each kernel is (3, 3). The kernel is a small matrix that is used for feature detection. A *ReLU* activation function layer is used for the *decoder* convolutional layers, *upsampling* is performed, and a *dropout* layer is included to avoid overfitting. Overall the model has 74,304 parameters.

#### 4.4.2. Performance Metrics

When reconstructing received noisy spectrograms, the objective function is, of course, to minimize the reconstruction error. The performance metrics of our DAE is the accuracy, defined by 1−Err, with

(7) $$Err=\frac{1}{\left|S\right|}\sum_{\ell =1}^{\left|S\right|}L\left(x_{\ell },z_{\ell }\right)$$

where the index *ℓ* runs over the set of training samples S and where L(x,z) is the MSE loss function.

#### 4.4.3. Results and Discussion for Signal Denoising

Besides accuracy, we assessed the reconstruction accuracy of our CDAE with different types of metrics for all channel scenarios and SNR levels.

Our CDAE achieves an average accuracy of above 77% within 500 epochs for all datasets under light to strong noise conditions, while SNR value varies from 0 to 20 dB across all channel scenarios. The lowest achieved accuracy is 76.79% for spectrograms under SNR 0 dB for *scenario 5* and *scenario 4*. The highest denoising accuracy for the CDAE is 79.96% for the dataset with SNR 20 dB and *scenario 4*. The radio signals with SNR 10 dB and *scenario 2* reaches 79.96% of testing accuracy with 295 epochs only. It was observed that the datasets of SNR 0 dB under the channel propagation model of *scenario 2* requires only 171 epochs to reach a 78% of accurate denoising.

With 77% average denoising accuracy, our CDAE successfully recovers the essential features for all IEEE 802.11 family (802.11a, IEEE 802.11n, IEEE 802.11ac, and IEEE 802.11ax) and unlicensed LTE signals. This encoding accuracy provides a robust foundation for protocol identification, as we discuss in more detail in the following subsection. The performance of our CDAE is uniform for all channel scenarios and SNR; this supports the notion of using a library of CDAEs to reconstruct the original $T{\left(x\right)}_{ij}$ regardless of the level of noise or the channel condition, or the type of the radio signal.

**Figure 6:**
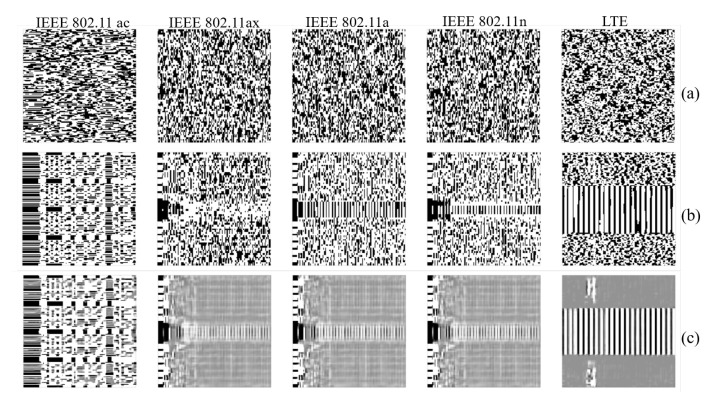
The spectrograms of five unlicensed radio signal for noise *scenario 1* with SNR 0 dB. Shown above: (**a**) the noisy spectrograms, (**b**) the clean spectrograms, and (**c**) the decoded spectrograms.

Figure 6 shows reconstructed spectrograms from different protocols in *scenario 1* with SNR 0 dB.

We remark that reconstructed spectrograms in Figure 6 are almost the same obtained across all channel scenarios for all the 25 datasets. Reconstructed spectrogram images clearly preserve the preamble for the WLAN IEEE 802.11 protocol and the subframes of the LTE signal. This could be related to the advantage of using convolutional layers, which preserves the spatial locality for input images. Our results emphasize the robustness of the CDAE architecture to reconstruct corrupted radio spectrogram, whatever the noise level or the channel propagation effect in the field.

### 4.5. Results and Discussion for Protocol Classification

Our experiment aims to measure the effectiveness of using CDAE compressed features as an input for a classifier to identify the unlicensed RATs, studying the performance of different Artificial Neural Network classifier architectures in identifying the protocols under different conditions. The different classifier configurations are summarized in Table 2. They are distinguished based on the number of layers, the use of convolutional or full connectivity layers (CNNs as opposed to ordinary *Multi-Layer Perceptrons* (MLPs)), and most importantly by the fact that they receive as inputs the original noisy images, or their compressed representation coming from our previously trained CDAE (this option is described in Table 2 as CDAE weights).

Notice that the structure of the input to the classifier changes depending on the use of the original noisy image or the CDAE representation: in the former case, the input size is (128,128,1), i.e., 128×128 a Boolean image of depth 1; in the latter case, it is (4,4,32), i.e., a set of 32 Boolean images each of size 4×4 (from the CDAE structure, one can already see that we imposed that the network learns 32 filters). Notice also that the input’s former representation has $2^{14}$ Boolean degrees of freedom, while our CDAE filter-based representation of the input only uses $2^{9}$ Boolean degrees of freedom.

The solution we put forward in this paper is reported in the last row of the table: it consists of a 24-layer CNN classifier receiving as input our CDAE representation. This pipeline outperforms other classifiers in the identification of the protocols (IEEE 802.11a, IEEE 802.11n, IEEE 802.11ax, IEEE 802.11ac, and unlicensed LTE) even under severe signal degradation (SNR is 0 dB) and for the different channel conditions (severe fading, multipath, NLOS, Doppler Shift, and all the previously described channel conditions). Moreover, most alternative models display a performance comparable to that of a random classifier (which, with 5 alternative classes, would display a 20% accuracy). A more detailed description follows.

The 4-layer MLP with and without CDAE, the 6-layer CNN classifier with and without CDAE, the 8-layer CNN classifier with and without CDAE, and the 13-layer CNN classifier without CDAE are stuck at a 20% accuracy across the whole considered range of channel scenarios and SNR levels. Overall, they perform as a random classifier. The CDAE representation with 24 layers, on the contrary, features an accuracy ranging from 60% to 100% for multiple protocol classification, depending on the noise and SNR values. CNN3 + AE performing better than CNN2 + AE suggests that adding one CNN layer helps to extract more features, which are useful for identification. Finding the dimensionality of the CNN which corresponds to the coarseness of data representation where the “right” features emerge is a classic problem, part of the CNN model’s hyperparameter tuning. We want to highlight that we explored both a lower number of layers and a higher number of layers. As it often happens, there is an optimal point in the complexity of a network that represents a good trade-off between bias and variance: the CNN3 + AE represents such a good trade-off. On the contrary, we found that the CNN2 + AE performance does not generalize well across all the datasets, while already the CNN4 + AE shows a clear sign of overfitting: we assume that increasing the number of layers thus further increasing the number of parameters would produce an even more apparent overfitting. Therefore, we did not experiment with CNN5 + AE, CNN6 + AE, and so on.

**Figure 7:**
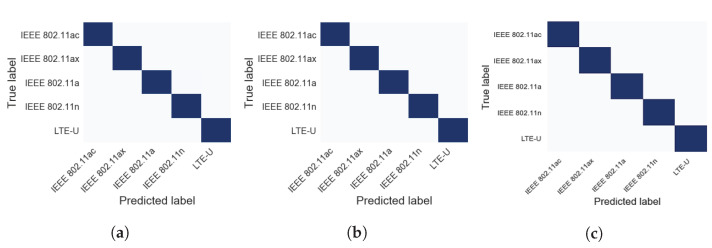
Confusion matrices for CNN3+AE in some representative SNR levels and channel scenarios: (**a**) SNR 5 dB and *scenario 5*; (**b**) SNR 15 dB and *scenario 3*; (**c**) SNR 20 dB and *scenario 2*.

Multiple performance metrics based on the confusion matrix, such as F-score (The F-score, also called the F1-score), recall, and precision, have also been used to evaluate the proposed model’s performance. A sample of the accuracy confusion matrices for the proposed classifier model across different datasets is presented in Figure 7.

Our proposed DL classifier model achieves an average accuracy of 95.44% for identifying a wireless technology that falls in the range of SNR 10 dB to 20 dB *across all the channel propagation effects*. The average accuracy of the wireless identification in intense noise environments considering the propagation effect scenarios’ effect is 82.55% for SNR range between 0 dB and 5 dB. The maximum achieved accuracy of our emitter classifier based on CDAE weights is 100%, and the average accuracy is 91% across all datasets.

## 5. Performance Comparison

In this section, we provide a full comparison of our pipeline’s performance with the one of traditional ML and DL-based models.

### 5.1. Comparison to Traditional ML Algorithms

In this section, we compare the performance of our *(CNN3 + AE)* pipeline to well-known ML algorithms such as *Support Vector Machine (SVM)* [71], *Random forest (RF)*, and *K-nearest neighbors (KNN)*. The performance of a shallow learner is also explored using a *one-dimensional Convolutional Neural Network (1D CNN).*

SVM is a supervised ML algorithm proposed in 1995 [72], where an optimal hyperplane is used to separate classes in the data space. SVM proved to be a successful method to attack a two-class classification task when extracting well-representative features from data. RF was first introduced by Tin Kam Ho [73]. It is an ensemble learning method for classification, where a multitude of decision trees are trained to output the label of the classes, and the predicted class is determined by majority voting. The KNN algorithm is a nonparametric, lazy learning algorithm that outputs each input’s class based on the majority of its nearest neighbors (proximity is measured by feature similarity) [74].

We tested these algorithms under all channel scenarios and SNR values to compare them to our proposed classifier. 1D CNN was chosen as a shallow benchmark learner as it enables frame-level investigation, and its use had been explored for audio recognition and Natural Language Processing (NLP). 1D CNN has been used with raw waveform and usually combined with a *Recurrent Neural Network* (RNN) in audio applications [75]. The convolution layer’s kernel size in our benchmark 1D CNN is set to 3, and 24 filters were used with a ReLU activation. The soft-max activation function was used to classify the protocols.

The input for SVM, KNN, RF, and 1D CNN consisted of the noisy spectrogram images of size 128×128, i.e., each image had 16,384 features; for the 1D CNN, this was shaped as a (16384,1) array. Each dataset was split to 80% for training, and the remaining 20% is for testing. In each training session, the data were shuffled, and 33% of it was used for cross-validation to tune the hyperparameters of the model whenever this could apply.

**Figure 8:**
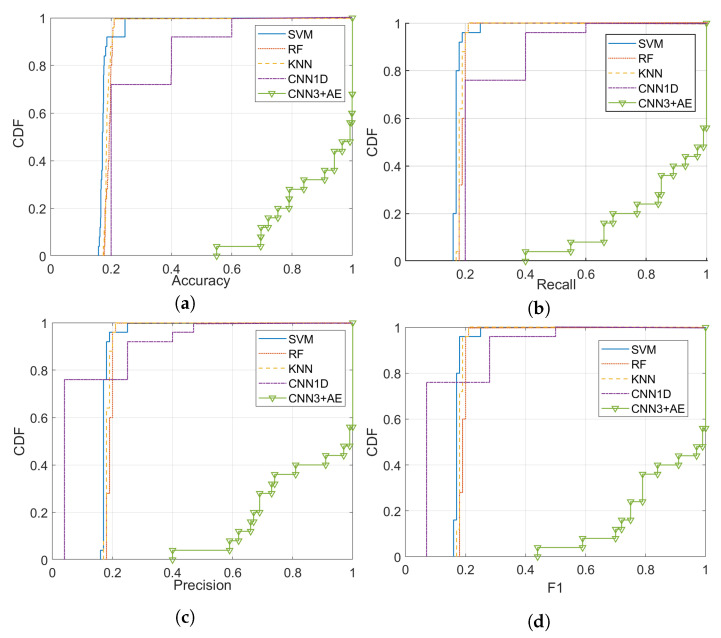
The overall performance cumulative distribution function (CDF) across a range of channel scenarios and signal-to-noise ratio (SNR) values: (**a**) Accuracy, (**b**) Recall, (**c**) Precision, and (**d**) F1.

Each classifier’s performance was evaluated using accuracy, recall, precision, and F-score [76]. Figure 8 shows the empirical cumulative distribution function (CDF) of the overall accuracy for the shallow classifier on our datasets across all the channel scenarios. The overall accuracy is the ratio of correctly predicted observation to the total observations. The accuracy of the SVM is the lowest, while KNN and RF achieve 20%. 1D CNN gives an accuracy of 20% in most of the datasets and 60% in a few datasets, depending on the channel scenario and SNR levels.

The recall (i.e., sensitivity) is shown in Figure 8b. The precision across all datasets under various noise conditions and SNR values is shown in Figure 8c. The overall CDF of F-score is illustrated in Figure 8d across all the 25 datasets.

**Figure 9:**
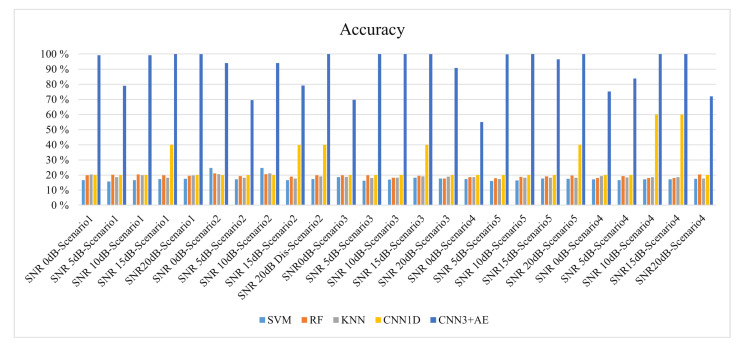
The overall accuracy for the traditional ML vs. the proposed approach across a range of channel scenarios and SNR values.

The performance of our (CNN3 + AE) is much higher than the other compared ML algorithms in terms of accuracy, recall, precision, and F1 score. The highest accuracy achieved by our developed model is 100% across many datasets such as a dataset with SNR 5 dB and *scenario 3*, 5 dB and *scenario 4*, and 5 dB and *scenario 4* as shown in Figure 9. The minimum accuracy is 55% for a dataset with the AWGN channel and SNR level equal to 0 dB (More features are recovered at 0 dB that were reported lost in [47].).

Our proposed DL classifier model achieves an average accuracy of 95.44% for the identification of a wireless technology that falls in the range of SNR 10 dB to 20 dB across all the channel and propagation effects. The average accuracy of the wireless identification in a strong noise environment considering the propagation effect scenarios’ effect is 82.55% for SNR range between 0 dB and 5 dB.

The overall average accuracy is 91%, calculated across all the SNR values and channel scenarios in the five unlicensed radio signals (across all datasets). Figure 9 depicts the results of the accuracy.

Based on our results, we conclude that the traditional supervised ML algorithms such as SVM, KNN, and RF did not perform well for protocol identification to classify unlicensed radio signals’ protocols such as IEEE 802.11a, IEEE 802.11n, IEEE 802.11ac, IEEE 802.11ax, and unlicensed LTE. Similar results are reported for SVM, KNN, and RF in [47], but it was not including the study of the propagation models in harsh environments.

### 5.2. Comparison to Benchmark DL Models

Deep CNNs are known to achieve good performance in image classification tasks. In this section, we report about the performance of some well-known deep CNN architectures, namely, the VGG [77], Inception [66], and ResNet [78] algorithms. The dataset for all benchmark DL architectures was split into 80% for the training set and 20% for the test set. During the training, 33% of the data was used for cross-validation to tune the DL classifier models’ hyperparameters. A max-pooling layer was used at the model’s output, and the soft-max layer was used to identify the class of the protocols.

In our experiments, we explored different DL architectures and evaluated their performance over unlicensed radio datasets at SNR 20 dB (light noise) and with AWGN to check their ability to identify the protocols in light noise environment conditions. Table 3 details the configuration and the results for different DL models, which are trained for SNR 20 dB with AWGN (scenario 5) dataset.

#### 5.2.1. Performance of VGG

VGG was developed for Large-Scale Image Recognition [77]. VGG uses a large number of small convolution filters. The size of filters usually is 3×3 and 1×1 with the stride of one. The number of filters in VGG depends on the depth of the VGG model. VGG has been used in modulation classification in [35] and shows good performance when combined with 1D CNN.

Table 3 summarizes the architectures of the two VGG classifiers which were trained and assessed. The input shape for the VGG3 and VGG16 classifiers was (128,128,1), i.e., a 128×128 Boolean image of depth 1. VGG16 was built using the Keras library, according to the architecture explained in [77].

Table 3 details the average precision, recall, F1-score, and accuracy for all the classifiers based on the VGG algorithm. Despite its depth, VGG16 cannot classify the protocols even in a very light noise scenario (SNR 20 dB with AWGN). The accuracy of VGG3 is 20%, equivalent to a random classifier for the five classes.

#### 5.2.2. Performance of Inception and GoogLeNet

The concept of *Inception* for very deep CNNs was introduced in [66] with the GoogLeNet model. The GoogLeNet model is based on a block of parallel convolutional layers with differently sized filters (1×1, 3×3, and 5×5) and a max-pooling layer (3×3). Then, the results of inception networks are composed by chaining.

In our experiments, we explored several architectures based on the inception model. The effect of the pooling layer on the output of the model was studied as well. The input shape for the Naive Inception model and Inception2 model is (128,128), while the spectrogram image is resized to (299,299) to fit the very deep convolutional Inception-V3 architecture, which is developed in [66]. The performance of Inception-V3 with global average pooling (GAP) in the output layer is better than other inception models as listed in Table 3. We can summarize the result by concluding that, despite the inception model’s depth, very deep inception networks feature low performance if one considers the associated cost of implementation.

#### 5.2.3. Performance of Residual Networks (ResNet)

Residual Networks (ResNets) are very deep convolutional network models proposed in 2016 [78]. ResNet is derived from the VGG deep convolutional networks by adding Residual blocks. Residual block consists of two convolutional layers. ReLU activation function is used for each convolutional layer. The output of each block is combined using a shortcut connection. ResNet-V2 is a modified residual network using Residual Inception Blocks, which provides good detection performance but is costlier than ResNet or Inception-V3 [65].

For our experiment, the effect of the output pooling layer is studied for ResNet-V2. The total parameters for each ResNet-V2 are stated in Table 3. The input images were resized to (299,299) to fit the developed architecture of ResNet-V2. The ResNet-V2-GAP classifier starts to improve the accuracy of classification of different protocols in the light noise scenario (SNR 20 dB with AWGN) in comparing with ResNet-V2-MAX.

The Inception-V3-GAP classifier can start identifying the protocols in the light noise scenario with the AWGN scenario, as indicated in Table 3. We also observed that the Inception-V3 with GAP layer performs better than ResNet-V2-GAP in classifying the spectrograms. However, the Inception-V3-GAP classification accuracy is low compared with our developed emitter classifier based on CDAE weights (CNN3 + AE). Inception-V3-GAP achieved 59.8% for SNR 20 dB in the AWGN channel scenario, which is considered a light noise condition in our experiment. Our CNN3 + AE model achieves 100% accuracy for the same dataset.

In summary, we observed that using DL models like inception networks, VGG blocks, or residual networks protocol identification does not improve the classification accuracy for protocols operating in unlicensed bands, even in light noise conditions such as SNR 20 dB and channel affected by AWGN only (scenario 5). ResNet-V2 and Inception-V3 with the GAP layer achieve very low accuracy even in a light noise than our developed model using DAE weights (CNN3 + AE). Our (CNN3 + AE) pipeline achieves 100% accuracy. Our (CNN3 + AE) has the lowest number of parameters compared to other DL models as highlighted in Table 3.

Complexity-wise, the number of Floating Point Operations (FLOs) expresses how computationally expensive a CNN model is. The FLOs of our proposed emitter model and other benchmark DL models were computed using the TensorFlow built-in profiler. Table 4 details the number of FLOs.

The number of FLOs for our proposed model (CNN3 + AE) is 68.4 million, which is much less than the number of FLOs for benchmark DL models. The number of FLOs for Inception-V3 and ResNet-V2 is 11.4 billion and 26.4 billion, respectively. The complexity is therefore very promising for the deployment of our proposed DL model in real-time applications.

We named our pipeline (CNN3 + AE) “**ConvAE**” DL model for protocol identification in unlicensed spectrum. **ConvAE** shows very high accuracy in identifying a range of radio access technologies in the unlicensed bands in harsh environments, which outperforms other well-known DL models in terms of accuracy and number of FLOs.

## 6. Conclusions

In this paper, we examined the use of DL to solve the coexistence problem between various communication technologies, achieving dynamic spectrum sharing and avoiding performance degradation. We studied our proposed DL method under various propagation channel models and very low SNR values. Specifically, we investigated using Convolutional Denoising Autoencoders (CDAEs) for reconstructing corrupted LTE and Wi-Fi spectrograms with the same carrier frequency under various channel scenarios and SNR values. We tested DL models to perform protocol identification for various IEEE 802.11 WLAN protocols and unlicensed LTE using CDAE weights. Our results show the benefit of performing CDAE before classifying the spectrograms under light to strong noise and different channel propagation conditions. Our proposed methodology for CDAE can reconstruct 77% of the corrupted signals sharing the same spectrum, while showing stable performance under severe noise conditions and propagation models. The achieved accuracy is sufficient to restore and preserve the preamble of the corrupted Wi-Fi 802.11 signals or the sub-frames of the transmitted unlicensed LTE signal. Furthermore, our methodology for protocol identification based on CDAE reduces the training parameters, learning time, and the number of FLOs compared to other DL models. Finally, our methodology significantly improves the average accuracy for protocol classification to 91% in identifying radio access technologies in the unlicensed bands compared to other well-known DL models such as VGG16, ResNet-v2, and Inception-V3.

## Figures and Tables

**Table 1:** Channel scenario based on LTE and IEEE 802.11 channel models.

Channel Scenario	Channel Condition for Unlicensed LTE	Channel Condition for IEEE 802.11
Scenario 1	LTE moving	Delay spread (model B) with d=3 m + No pathloss
Scenario 2	LTE moving	Delay spread (model B) with d=10 m + pathloss
Scenario 3	LTE fading channel	Delay spread (model B) with d=30 m + pathloss
Scenario 4	LTE moving with no fading	Doppler
Scenario 5	AWGN	AWGN

**Table 2:** Classifier configurations. The proposed classifier architecture is highlighted.

Classifier Name	Type	Number of Layers
MLP	MLP	4
MLP + AE	MLP + CDAE weights	15
CNN1	CNN	6
CNN1 + AE	CNN + CDAE weights	17
CNN2	CNN	8
CNN2 + AE	CNN + CDAE weights	19
CNN3	CNN	13
**CNN3 + AE**	CNN+ CDAE weights	24

**Table 3:** Comparison to Benchmark DL Models using dataset of SNR 20 dB with AWGN (scenario 5). The highest performance number is highlighted. The lowest number of the model parameters is highlighted.

Architecture Name	Architectures	Output Pooling Layer	# Parameters	Precision	Recall	F1-Score	Accuracy
VGG3	3 VGG blocks	Max pooling layer	3,269,605	4%	20%	7%	20%
VGG16	16 VGG blocks	Max pooling layer	14,716,101	4%	20%	7%	20%
Naive Inception	1 Inception block	Max pooling layer	7,947,461	4%	20%	7%	20%
Inception2	2 Inception blocks	Max pooling layer	10,572,293	4%	20%	7%	20%
Inception-V3-MAX	Inception blocks [66]	Max pooling layer	25,997,285	4%	20%	7%	20%
Inception-V3-GAP	Inception blocks in [66]	Global average pooling layer	21,812,453	40%	60%	47%	59.8%
ResNet-V2-MAX	Residual Networks	Max pooling layer	57,482,661	4%	20%	7%	20%
ResNet-V2-GAP	Residual Networks	Global average pooling layer	54,343,845	11%	21%	14%	21%
**The proposed DL Model: CNN3 + AE**	CNN + CDAE weights	Max pooling layer	**51,401**	**100%**	**100%**	**100%**	**100%**

**Table 4:** Floating point operations for the different models. The smallest number is highlighted.

Model	FLOs
**CNN3 + AE**	**68.4 Millions**
Inception-V3	11.4 Billions
ResNet-V2	26.4 Billions

## Data Availability

The data presented in this study are available on request from the corresponding author.

## Appendix A. Simulation Setup for the IEEE 802.11 Family

In our research, we focus on WLAN devices that are operating in the 5 GHz ISM band to study the performance of ML in identifying IEEE 802.11 protocols and LTE signals. For this reason, IEEE 802.11a, IEEE 802.11n, IEEE 802.11ac (Wi-Fi 5), and IEEE 802.11ax (Wi-Fi6) were selected.

### Appendix A.1. Physical Layer for IEEE 802.11

A detailed system analysis is required to implement WLAN systems. The IEEE 802.11 WLAN standard provides several PHYsical layers (PHY) options specified for each IEEE 802.11 standard in channel bandwidth, modulation, and spreading spectrum techniques. The IEEE 802.11a PHY adopts OFDM, IEEE 802.11n is based on high-throughput (HT)-OFDM. Very high throughput (VHT)-OFDM is the physical layer for IEEE 802.11ac, and high-efficiency (HE)-OFDM is the PHY layer for IEEE 802.11ax. Table A1 summarizes the simulation parameters for the PYH layer of IEEE protocols which were selected for our analysis: IEEE 802.11a, IEEE 802.11n, IEEE 802.11ac (*Wi-Fi5*), and IEEE 802.11ax (*Wi-Fi6*).

**Table A1:** WLAN standards simulation parameters.

Parameters	IEEE 802.11a	IEEE 802.11n	IEEE 802.11ax (*Wi-Fi6*)	IEEE 802.11ac (*Wi-Fi5*)
Modulation	OFDM	HT-OFDM	HE-OFDM	VHT-OFDM
Channel bandwidth	20 MHz	20 MHz	20 MHz	80 MHz
Preamble	Long	Long	Long	Long
PSDULength	1000	1000	100	1035
Number of transmitted antennas	1	1	1	1
Modulation and coding scheme	0 “BPSK (Binary phase shift keying)”	0 “BPSK”	0 “BPSK”	0 “BPSK”
Spatial Mapping Matrix	N/A	1	1	1
Spatial Mapping	N/A	Direct	Direct	Direct
Channel Coding	N/A	BCC (Binary convolutional coding)	LDPC (Low-density parity-check coding)	BCC
Number Space Time Streams	N/A	1	1	1

### Appendix A.2. Channel Conditions for IEEE 802.11 Protocols

The channel medium between the transmitter and receiver in IEEE 802.11 protocols is affected by multiple channel propagation models and SNR levels. A set of channel models was designed and studied thoroughly to provide sufficient channel models for IEEE 802.11 PHY layer simulation and performance testing for IEEE 802.11n WLAN [79], 802.11 ac [80], 802.11ax [81], and 802.11a [82]. Each channel model is designed for a certain environment. Each channel has a certain number of clusters, where each cluster includes a set of taps. The number of clusters, number of taps, the value of Root mean square (RMS) delay ($\sigma_{RMS}$), the maximum delay ($\sigma_{Max}$), and the standard deviation σ value of shadow fading are all characterized in each model in case of Line-of-Sight (LOS) and NLOS conditions.

Moreover, there are two key signal propagation models for WLAN channel modeling: (1) Large-scale propagation (or large-scale path loss) and (2) small-scale fading.

For large scale path loss, the considered path loss model L(d) for indoor WLAN channel is based on the following equation:

(A1) $$L\left(d\right)=\left\{\begin{array}{c} L_{FS}\left(d\right)\;\;\;\;\;\mathrm{if}\;d\leq d_{BP}\\ L_{FS}\left(d_{BP}\right)+3.5log10\left(d/d_{BP}\right)\;\mathrm{if}\;d>d_{BP} \end{array}\right.$$

where *d* is the distance between the transmitter and receiver, $L_{FS}$ is the free space loss (log-distance model) for the slope of 2 for up to break-point distance $d_{BP}$ and 3.5 after $d_{BP}$. The $d_{BP}$ for our channel model is 5 m. Generally, the breakpoint distance is assumed to be the boundary for LOS and NLOS conditions [79]:

(A2) $$L_{FS}\left(d\right)=-10log_{10}\left(\frac{G_{t}G_{r}\lambda^{2}}{{\left(4\pi d\right)}^{2}}\right)$$

where $G_{t}$ and $G_{r}$ are the transmitter and the receiver gain, which we assume equal to 1, λ is the wavelength of the transmitted carrier frequency $f_{c}$ at speed of radio frequency (RF) signal $\lambda =\frac{\nu }{f_{c}}$. For our implementation, the RF signal speed ν (approximately the speed of light) is equal to $\nu =c=3\times 10^{8}$ m/sec.

(A3) $$L_{FS}\left(d\right)=10log_{10}\left(\frac{{\left(4\pi d\right)}^{2}}{\lambda^{2}}\right)$$

(A4) $$L_{FS}\left(d\right)=47dB+2.10log_{10}\left(d\right)$$

In our simulations, we consider the channel path loss model in an indoor and open environment. Thus, *Model B* delay profile is selected as a channel model for IEEE 802.11ac, 802.11ax, and 802.11n. *Model B* represents a typical sizable open space and office environments with NLOS conditions and 100 ns RMS delay spread for IEEE 802.11. The parameters used to model path loss to provide sufficient channel models for IEEE 802.11n PHY layer simulation are detailed in [79], for IEEE 802.11ac PHY layer in [80], and for IEEE 802.11ax PHY layer in [83]. The channel path loss model for IEEE 802.11a is simulated as a Rayleigh channel model, with a sample rate of $f_{s}=20$ MHz, and the carrier frequency $f_{c}$ is 5.25 GHz.

The shadow fading (also known as log-normal shadowing) is also considered in our analysis. It is modeled with a zero-mean Gaussian random variable *x* and a standard deviation σ, and added to the path loss model as given by

(A5) $$L\left(d\right)=\left\{\begin{array}{c} L_{FS}\left(d\right)+X_{\sigma }\;\;\;\;\;\;\;\;\mathrm{if}\;d\leq d_{BP}\\ L_{FS}\left(d_{BP}\right)+3.5log10\left(d/d_{BP}\right)+X_{\sigma }\;\mathrm{if}\;d>d_{BP} \end{array}\right.$$

where $X_{\sigma }$ is the random variable and the value of σ differs before and after the break-point distance $d_{BP}$ as described in [79].

For our analysis and propagation models, small-scale fading [84] is also considered. Small-scale fading causes signal distortion for the transmitted WLAN signal. Various physical aspects induce small-scale fading effects such as mobile speed, multipath propagation, surrounding objects motion, and the transmission bandwidth of the signal [84]. The motion of the people around the environment causes Doppler spread $f_{d}$, defined as follows:

(A6) $$f_{d}=\frac{\nu_{o}}{\lambda }$$

where $\nu_{o}$ is the environmental speed. For our experiment, where $f_{c}$ is 5.25 GHz, the wavelength $\lambda =\frac{3\times 10^{8}\;m/s}{5.25\times 10^{9}\;\mathrm{Hz}}=0.0571$ m. $f_{d}$ is calculated and encountered in the simulated environment for the received signal for each WLAN IEEE 802.11. The typical walking speeds for indoor environments is approximately 1.2 km/h (0.333 m/s) [79].

(A7) $$f_{d}=\frac{0.333m/s}{0.0571m}=5.832\;\mathrm{Hz}$$

The worst-case frequency shift known as Doppler spread is around 5.8 Hz for WLAN packets.

### Appendix A.3. Received Signal for the IEEE 802.11 Protocols

The received signals R(x) for all protocols are captured under various channel propagation conditions H(x) as summarized in Table A2. Each IEEE 802.11 standard is simulated under five propagation channels H(x) independently. Each channel H(x) is measured with a range of SNR levels. Three values for the T-R separation *d* between the transmitter and receiver are incorporated to explore the propagation path loss model effect with small T-R separation d=3 m, medium T-R separation d=10 m, and large T-R separation *d* = 30 m. Additive White Gaussian Noise (AWGN) is added at different SNR levels n(x) for all channel propagation models. Moreover, the receiver noise is added to the received signal R(x) with a Noise Figure (NF) equals 9 dB.

**Table A2:** The channel propagation conditions H(x) for IEEE 802.11 protocols.

Channel 1	Delay spread (model B) with d=3 m + pathloss
Channel 2	Delay spread (model B) with d=10 m + pathloss
Channel 3	Delay spread (model B) with d=30 m + No pathloss
Channel 4	Doppler effect
Channel 5	Only AWGN

## Appendix B. Simulation Setup for LTE

Unlicensed LTE is based on the 3GPP Release 12 LTE technology to be used in the unlicensed spectrum. LTE is used as a secondary cell within the LTE carrier aggregation framework anchored by a licensed primary cell. In our simulation, the downlink system for LTE is considered.

### Appendix B.1. Physical Layer for LTE

The downlink physical layer of LTE is based on orthogonal frequency-division multiple access (OFDMA).

For our simulation, LTE is built based on a single transmitter and a single receiver. The LTE frame consists of 10 subframes that are individually generated. The frame is OFDM modulated. The LTE frame structure for the downlink is Frequency Division Duplexing (FDD). Each user is assigned to a different time/frequency Resource Block (RB). The simulation parameters of evolved NodeB (eNB) in LTE-subframe are presented in Table A3.

**Table A3:** The parameters for the physical downlink layer of eNB-LTE.

Number of resource blocks	6
Number of transmit antennas	1
Duplex mode	FDD
Cyclic prefix	Normal

### Appendix B.2. LTE Channel Conditions

In LTE, RB is assigned to user equipment (UE) from eNB in the downlink channel. The LTE signal suffers from signal degradation due to a dynamic change of the distance between eNB and UE, radio power level, noise level, and multipath fading effects.

The downlink unlicensed-LTE signal is affected by the following: (1) LTE moving channel of propagation conditions which implements *scenario 1* for an extended typical urban environment using an (ETU200) Rayleigh fading model with 200 Hz Doppler shift and changing delays, (2) LTE moving channel with *scenario 2* which represents a single non-fading model as specified in [85,86], (3) LTE fading channel for a multipath fading MIMO channel propagation conditions using a Generalized Method of Exact Doppler Spread (GMEDS) for a Rayleigh fading model type [87], and (4) AWGN.

In our simulation, the LTE radio signals are exposed across a range of light to serve noise conditions where the SNR ranges from 0 dB to 20 dB. Besides, the LTE downlink signal is affected by three main propagation channel models and the AWGN. The downlink LTE signal is affected by (1) LTE moving channel of propagation conditions which implements *scenario 1*, (2) LTE moving channel with *scenario 2*, (3) LTE fading channel, and (4) AWGN. The LTE moving propagation scenarios are implemented as specified in TS 36.104, Annex B.4 [85]. The used parameters of the LTE fading channel model are specified in [87]. A summary of the implemented channel conditions for unlicensed-LTE is presented in Table A4.

**Table A4:** The channel conditions for unlicensed-LTE.

Channel 1	LTE moving—*Scenario 1*
Channel 2	LTE moving (No fading)—*Scenario 2*
Channel 3	LTE fading
Channel 4	AWGN

### Appendix B.3. LTE Received Signal

The received LTE signals in UE suffer from degradation due to the channel propagation models and SNR levels in the reception. AWGN is included in all LTE channel models. Four downlink channel environments are simulated to the LTE R(x). Each channel model H(x) is generated at SNR values ranges from 0 to 20 dB. The receiver noise with an NF equals 9 dB is added to the received LTE signal R(x).

We study protocol identification’s effect under all the mentioned channel propagation conditions for WLAN IEEE 802.11 and unlicensed-LTE. A summary of the implemented propagation model scenarios for unlicensed-LTE signals and IEEE 802.11 channel models is described in Table 1.

## References

[1] Chen B., Chen J., Gao Y., Zhang J. (2016). Coexistence of LTE-LAA and Wi-Fi on 5 GHz with corresponding deployment scenarios: A survey. IEEE Commun. Surv. Tutor..

[2] Bojović B., Giupponi L., Ali Z., Miozzo M. (2019). Evaluating unlicensed LTE technologies: LAA vs LTE-U. IEEE Access.

[3] Naik G., Park J.M., Ashdown J., Lehr W. (2020). Next Generation Wi-Fi and 5G NR-U in the 6 GHz Bands: Opportunities & Challenges. IEEE Access.

[4] Jiang C., Zhang H., Ren Y., Han Z., Chen K.C., Hanzo L. (2017). Machine learning paradigms for next-generation wireless networks. IEEE Wirel. Commun..

[5] Hessar M., Najafi A., Iyer V., Gollakota S. TinySDR: Low-Power SDR Platform for Over-the-Air Programmable IoT Testbeds. Proceedings of the 17th USENIX Symposium on Networked Systems Design and Implementation (NSDI 20).

[6] Goodfellow I., Bengio Y., Courville A., Bengio Y. (2016). Deep Learning.

[7] Bengio Y., Courville A., Vincent P. (2013). Representation Learning: A Review and New Perspectives. IEEE Trans. Pattern Anal. Mach. Intell..

[8] Simeone O. (2018). A very brief introduction to machine learning with applications to communication systems. IEEE Trans. Cogn. Commun. Netw..

[9] O’Shea T.J., Corgan J., Clancy T.C. Convolutional radio modulation recognition networks. Proceedings of the International Conference on Engineering Applications of Neural Networks.

[10] Jagannath J., Polosky N., O’Connor D., Theagarajan L.N., Sheaffer B., Foulke S., Varshney P.K. Artificial neural network based automatic modulation classification over a software defined radio testbed. Proceedings of the 2018 IEEE International Conference on Communications (ICC).

[11] Schmidt M., Block D., Meier U. Wireless interference identification with convolutional neural networks. Proceedings of the 2017 IEEE 15th International Conference on Industrial Informatics (INDIN).

[12] Kulin M., Kazaz T., Moerman I., De Poorter E. (2018). End-to-end learning from spectrum data: A deep learning approach for wireless signal identification in spectrum monitoring applications. IEEE Access.

[13] O’Shea T., Hoydis J. (2017). An introduction to deep learning for the physical layer. IEEE Trans. Cogn. Commun. Netw..

[14] Ye H., Li G.Y., Juang B.H. (2017). Power of deep learning for channel estimation and signal detection in OFDM systems. IEEE Wirel. Commun. Lett..

[15] O’Shea T.J., Erpek T., Clancy T.C. (2017). Deep learning based MIMO communications. arXiv.

[16] Yan X., Long F., Wang J., Fu N., Ou W., Liu B. Signal detection of MIMO-OFDM system based on auto encoder and extreme learning machine. Proceedings of the 2017 International Joint Conference on Neural Networks (IJCNN).

[17] Ye H., Li G.Y., Juang B.H.F., Sivanesan K. Channel agnostic end-to-end learning based communication systems with conditional GAN. Proceedings of the 2018 IEEE Globecom Workshops (GC Wkshps).

[18] Cui W., Shen K., Yu W. (2019). Spatial deep learning for wireless scheduling. IEEE J. Sel. Areas Commun..

[19] Zhang C., Patras P., Haddadi H. (2019). Deep learning in mobile and wireless networking: A survey. IEEE Commun. Surv. Tutor..

[20] Almazrouei E., Gianini G., Almoosa N., Damiani E. What Can Machine Learning Do for Radio Spectrum Management?. Proceedings of the 16th ACM Symposium on QoS and Security for Wireless and Mobile Networks, Q2SWinet’20.

[21] Chen M., Challita U., Saad W., Yin C., Debbah M. (2019). Artificial Neural Networks-Based Machine Learning for Wireless Networks: A Tutorial. IEEE Commun. Surv. Tutor..

[22] Gündüz D., de Kerret P., Sidiropoulos N.D., Gesbert D., Murthy C.R., van der Schaar M. (2019). Machine Learning in the Air. IEEE J. Sel. Areas Commun..

[23] Farsad N., Goldsmith A. (2018). Neural Network Detection of Data Sequences in Communication Systems. IEEE Trans. Signal Process..

[24] Rajendran S., Calvo-Palomino R., Fuchs M., Van den Bergh B., Cordobes H., Giustiniano D., Pollin S., Lenders V. (2018). Electrosense: Open and Big Spectrum Data. IEEE Commun. Mag..

[25] Shi L., Bahl P., Katabi D. Beyond Sensing: Multi-GHz Realtime Spectrum Analytics. Proceedings of the 12th USENIX Symposium on Networked Systems Design and Implementation (NSDI 15).

[26] Terry B.C., Orange A., Patwari N., Kasera S., Van Der Merwe J. Spectrum Monitoring and Source Separation in POWDER. Proceedings of the 14th International Workshop on Wireless Network Testbeds, Experimental Evaluation & Characterization, WiNTECH’20.

[27] Zeng Y., Chandrasekaran V., Banerjee S., Giustiniano D. A Framework for Analyzing Spectrum Characteristics in Large Spatio-Temporal Scales. Proceedings of the 25th Annual International Conference on Mobile Computing and Networking, MobiCom’19.

[28] Zheleva M.Z., Chandra R., Chowdhery A., Garnett P., Gupta A., Kapoor A., Valerio M. (2018). Enabling a Nationwide Radio Frequency Inventory Using the Spectrum Observatory. IEEE Trans. Mob. Comput..

[29] Feng Q., Zhang Y., Li C., Dou Z., Wang J. (2017). Anomaly detection of spectrum in wireless communication via deep auto-encoders. J. Supercomput..

[30] O’Shea T.J., Clancy T.C., McGwier R.W. (2016). Recurrent Neural Radio Anomaly Detection. arXiv.

[31] Das R., Gadre A., Zhang S., Kumar S., Moura J.M.F. A Deep Learning Approach to IoT Authentication. Proceedings of the 2018 IEEE International Conference on Communications (ICC).

[32] Riyaz S., Sankhe K., Ioannidis S., Chowdhury K. (2018). Deep Learning Convolutional Neural Networks for Radio Identification. IEEE Commun. Mag..

[33] Brik V., Banerjee S., Gruteser M., Oh S. Wireless device identification with radiometric signatures. Proceedings of the 14th ACM International Conference on Mobile Computing and Networking.

[34] O’Shea T.J., West N., Vondal M., Clancy T.C. Semi-supervised radio signal identification. Proceedings of the 2017 19th International Conference on Advanced Communication Technology (ICACT).

[35] O’Shea T.J., Roy T., Clancy T.C. (2018). Over-the-air deep learning based radio signal classification. IEEE J. Sel. Top. Signal Process..

[36] O’Shea T.J., Corgan J., Clancy T.C. Unsupervised representation learning of structured radio communication signals. Proceedings of the 2016 First International Workshop on Sensing, Processing and Learning for Intelligent Machines (SPLINE).

[37] Peng S., Jiang H., Wang H., Alwageed H., Zhou Y., Sebdani M.M., Yao Y.D. (2018). Modulation Classification Based on Signal Constellation Diagrams and Deep Learning. IEEE Trans. Neural Netw. Learn. Syst..

[38] Rajendran S., Meert W., Giustiniano D., Lenders V., Pollin S. (2018). Deep Learning Models for Wireless Signal Classification With Distributed Low-Cost Spectrum Sensors. IEEE Trans. Cogn. Commun. Netw..

[39] Hu S., Pei Y., Liang P.P., Liang Y. (2020). Deep Neural Network for Robust Modulation Classification Under Uncertain Noise Conditions. IEEE Trans. Veh. Technol..

[40] Tan J., Zhang L., Liang Y.C., Niyato D. (2020). Intelligent sharing for LTE and WiFi Systems in Unlicensed Bands: A Deep Reinforcement Learning Approach. IEEE Trans. Commun..

[41] Selim A., Paisana F., Arokkiam J.A., Zhang Y., Doyle L., DaSilva L.A. Spectrum Monitoring for Radar Bands Using Deep Convolutional Neural Networks. Proceedings of the GLOBECOM 2017—2017 IEEE Global Communications Conference.

[42] Nika A., Zhang Z., Zhou X., Zhao B.Y., Zheng H. Towards Commoditized Real-Time Spectrum Monitoring. Proceedings of the 1st ACM Workshop on Hot Topics in Wireless, HotWireless’14.

[43] Rayanchu S., Patro A., Banerjee S. Airshark: Detecting Non-WiFi RF Devices Using Commodity WiFi Hardware. Proceedings of the 2011 ACM SIGCOMM Conference on Internet Measurement Conference.

[44] Hong S.S. DOF: A Local Wireless Information Plane. Proceedings of the ACM SIGCOMM 2011 Conference, SIGCOMM’11.

[45] Guddeti Y., Subbaraman R., Khazraee M., Schulman A., Bharadia D. Sweepsense: Sensing 5 ghz in 5 milliseconds with low-cost radios. Proceedings of the 16th {USENIX} Symposium on Networked Systems Design and Implementation ({NSDI} 19).

[46] O’Shea T.J., Roy T., Erpek T. Spectral detection and localization of radio events with learned convolutional neural features. Proceedings of the 2017 25th European Signal Processing Conference (EUSIPCO).

[47] Bitar N., Muhammad S., Refai H.H. Wireless technology identification using deep Convolutional Neural Networks. Proceedings of the 2017 IEEE 28th Annual International Symposium on Personal, Indoor, and Mobile Radio Communications (PIMRC).

[48] Abadi M., Andersen D.G. (2016). Learning to protect communications with adversarial neural cryptography. arXiv.

[49] Grunau S., Block D., Meier U. Multi-Label Wireless Interference Classification with Convolutional Neural Networks. Proceedings of the 2018 IEEE 16th International Conference on Industrial Informatics (INDIN).

[50] Behura S., Kedia S., Hiremath S.M., Patra S.K. (2020). WiST ID -Deep Learning-Based Large Scale Wireless Standard Technology Identification. IEEE Trans. Cogn. Commun. Netw..

[51] Danev B., Capkun S. Transient-Based Identification of Wireless Sensor Nodes. Proceedings of the 2009 International Conference on Information Processing in Sensor Networks, IPSN’09.

[52] Krizhevsky A., Sutskever I., Hinton G.E. (2017). ImageNet Classification with Deep Convolutional Neural Networks. Commun. ACM.

[53] Tran T.X., Hajisami A., Pandey P., Pompili D. (2017). Collaborative Mobile Edge Computing in 5G Networks: New Paradigms, Scenarios, and Challenges. IEEE Commun. Mag..

[54] Shen Y., Ferdman M., Milder P. Maximizing CNN accelerator efficiency through resource partitioning. Proceedings of the 2017 ACM/IEEE 44th Annual International Symposium on Computer Architecture (ISCA).

[55] Alizadeh Vahid K., Prabhu A., Farhadi A., Rastegari M. Butterfly Transform: An Efficient FFT Based Neural Architecture Design. Proceedings of the 2020 IEEE/CVF Conference on Computer Vision and Pattern Recognition (CVPR).

[56] Sze V., Chen Y., Yang T., Emer J.S. (2017). Efficient Processing of Deep Neural Networks: A Tutorial and Survey. Proc. IEEE.

[57] Mertins A., Mertins D.A. (1999). Signal Analysis: Wavelets, Filter Banks, Time-Frequency Transforms and Applications.

[58] Guddeti Y., Subbaraman R., Khazraee M., Schulman A., Bharadia D. (2020). Towards Low-Cost, Ubiquitous High-Time Resolution Sensing for Terrestrial Spectrum. Getmobile Mob. Comp. Comm..

[59] Vincent P., Larochelle H., Lajoie I., Bengio Y., Manzagol P.A. (2010). Stacked denoising autoencoders: Learning useful representations in a deep network with a local denoising criterion. J. Mach. Learn. Res..

[60] Mio C., Gianini G. Signal reconstruction by means of Embedding, Clustering and AutoEncoder Ensembles. Proceedings of the 2019 IEEE Symposium on Computers and Communications (ISCC).

[61] Lee D., Choi S., Kim H.J. (2018). Performance evaluation of image denoising developed using convolutional denoising autoencoders in chest radiography. Nucl. Instrum. Methods Phys. Res. Sect. Accel. Spectrom. Detect. Assoc. Equip..

[62] Almazrouei E., Gianini G., Almoosa N., Damiani E. A Deep Learning Approach to Radio Signal Denoising. Proceedings of the 2019 IEEE Wireless Communications and Networking Conference Workshop (WCNCW).

[63] Almazrouei E., Gianini G., Mio C., Almoosa N., Damiani E. Using AutoEncoders for Radio Signal Denoising. Proceedings of the 15th ACM International Symposium on QoS and Security for Wireless and Mobile Networks.

[64] Nandi A.K., Azzouz E.E. (1998). Algorithms for automatic modulation recognition of communication signals. IEEE Trans. Commun..

[65] Szegedy C., Ioffe S., Vanhoucke V., Alemi A.A. Inception-v4, inception-resnet and the impact of residual connections on learning. Proceedings of the Thirty-First AAAI Conference on Artificial Intelligence.

[66] Szegedy C., Liu W., Jia Y., Sermanet P., Reed S., Anguelov D., Erhan D., Vanhoucke V., Rabinovich A. Going deeper with convolutions. Proceedings of the IEEE Conference on Computer Vision and Pattern Recognition.

[67] Ji S., Xu W., Yang M., Yu K. (2012). 3D convolutional neural networks for human action recognition. IEEE Trans. Pattern Anal. Mach. Intell..

[68] Wang X., Mao S., Gong M.X. (2017). A survey of LTE Wi-Fi coexistence in unlicensed bands. Getmob. Mob. Comput. Commun..

[69] Bellalta B. (2016). IEEE 802.11 ax: High-efficiency WLANs. IEEE Wirel. Commun..

[70] López-Pérez D., Garcia-Rodriguez A., Galati-Giordano L., Kasslin M., Doppler K. (2019). IEEE 802.11 be Extremely High Throughput: The Next Generation of Wi-Fi Technology Beyond 802.11 ax. IEEE Commun. Mag..

[71] Tsang I.W., Kwok J.T., Cheung P.M. (2005). Core vector machines: Fast SVM training on very large data sets. J. Mach. Learn. Res..

[72] Cortes C., Vapnik V. (1995). Support-vector networks. Mach. Learn..

[73] Ho T.K. (1998). The random subspace method for constructing decision forests. IEEE Trans. Pattern Anal. Mach. Intell..

[74] Altman N.S. (1992). An introduction to kernel and nearest-neighbor nonparametric regression. Am. Stat..

[75] Lim H., Park J., Han Y. Rare sound event detection using 1D convolutional recurrent neural networks. Proceedings of the Detection and Classification of Acoustic Scenes and Events 2017 Workshop.

[76] Powers D.M. (2011). Evaluation: From precision, recall and F-measure to ROC, informedness, markedness and correlation. arXiv.

[77] Simonyan K., Zisserman A. (2014). Very deep convolutional networks for large-scale image recognition. arXiv.

[78] He K., Zhang X., Ren S., Sun J. Deep residual learning for image recognition. Proceedings of the IEEE Conference on Computer Vision and Pattern Recognition.

[79] Erceg V., Schumacher L., Kyritsi P. (2004). IEEE 802.11 Document 03/940r4 (TGn Channel Models).

[80] Breit G., Sampath H., Vermani S. (2009). TGac channel model addendum support material. Mentor IEEE, Doc IEEE 802.11-09/06/0569r0.

[81] Liu J., Porat R., Jindal N. (2014). IEEE 802.11 ax channel model document. Wireless LANs, Rep. IEEE.

[82] Doufexi A., Armour S., Butler M., Nix A., Bull D., McGeehan J., Karlsson P. (2002). A comparison of the HIPERLAN/2 and IEEE 802.11 a wireless LAN standards. IEEE Commun. Mag..

[83] Jianhan Liu R.P. (2014). TGax Channel Model. IEEE 802.11-14/0882r4.

[84] Rappaport T.S. (1996). Wireless Communications: Principles and Practice.

[85] (2011). User Equipment (UE) Conformance Specification Radio.

[86] Lte E. (2009). Evolved Universal Terrestrial Radio Access (e-Utra); Base Station (bs) Radio Transmission and Reception (3gpp ts 36.104 Version 8.6. 0 Release 8), July 2009.

[87] Patzold M., Wang C.X., Hogstad B.O. (2009). Two new sum-of-sinusoids-based methods for the efficient generation of multiple uncorrelated Rayleigh fading waveforms. IEEE Trans. Wirel. Commun..

